# Nonreciprocal spontaneous parametric process

**DOI:** 10.1038/s41377-025-01844-8

**Published:** 2025-05-19

**Authors:** Changbiao Li, Jiaqi Yuan, Ruidong He, Jiawei Yu, Yanpeng Zhang, Min Xiao, Keyu Xia, Zhaoyang Zhang

**Affiliations:** 1 Key Laboratory for Physical Electronics and Devices of the Ministry of Education & Shaanxi Key Lab of Information Photonic Technique, School of Electronic Science and Engineering, Faculty of Electronic and Information Engineering, Xi’an Jiaotong University, Xi’an, 710049 China; 2 Department of Physics, University of Arkansas, Fayetteville, AR 72701 USA; 3 College of Engineering and Applied Sciences, National Laboratory of Solid State Microstructures, and School of Physics, Nanjing University, Nanjing, 210093 China

**Keywords:** Nonlinear optics, Atom optics

## Abstract

Mediated by the interactions with quantum vacuum fields, a probe laser field propagating in a nonlinear optical medium can generate new pair of light fields over a broad spectral range via spontaneous parametric process. Such process is inherently independent of the incident direction of light and reciprocal thus far, due to the direction-independent field-vacuum interactions. In this work, we experimentally demonstrate within sodium atomic vapors that such spontaneous parametric process can be nonreciprocal by unidirectionally coupling it to another pumped four-wave mixing process. Thanks to the broad bandwidth of the spontaneous parametric process, in combination with the Doppler and power-induced broadening of atomic energy levels, we achieve optical isolation with isolation ratio >25 dB over a bandwidth larger than 100 GHz. Considering that both spontaneous parametric processes and the pumped four-wave mixing have been realized in diverse solid photonic platforms, the demonstrated concept can motivate further explorations in the design of integrated magnetic-free broadband optical nonreciprocity via the interactions between nonlinear optical processes.

## Introduction

A basic concept in classical electromagnetic theory based on the Maxwell’s equations is that the electromagnetic field vanishes in a “null” closing space in which internal sources and external driving are absent. In stark contrast, quantum optics reveals that an empty space even without any real excitations is filled with non-zero quantum fluctuation, which is known as the quantum vacuum field and has been directly observed with a superconducting atom^1–3^. As one of the deepest fundamental properties of nature, quantum vacuum field plays critically important roles for understanding many fundamental quantum processes such as spontaneous decay of atoms^4^, the Casimir effect^5,6^, and cavity quantum electrodynamics^7^. Beside fundamental physics, quantum vacuum fields can efficiently trigger the generation of new light fields via the third-order nonlinear processes, such as the spontaneous parametric process or the pumped four-wave mixing (FWM)^8^, when the involved fields simultaneously meet the phase-matching condition (PMC, governing the momentum conservation) and energy conservation. The spontaneous parametric process can produce a pair of photons with two probe photons annihilated, while the pumped FWM process can give rise to a new light beam under three-beam excitation^9^.

Since the required PMC is crucially dependent on the directions of the involved driving fields, the pumped FWM can occur unidirectionally, indicating a nonreciprocal optical process^10^. Optical nonreciprocity breaking the Lorentz reciprocity can perform optical isolation by enforcing one-way propagation of light. Such property plays vital roles in laser protection, optical and integrated photonic technologies^11^, and even quantum information processing^12–16^. It’s worth mentioning that nonreciprocal optical devices (NORDs) based on wave conversion attract intensive attentions because they are inherently compatible with integrated photonic technologies. *However*, *the spontaneous parametric process is by far reciprocal because of the time-reversal and spatial inversion symmetries of quantum fluctuation and its interaction with a coherent probe field*. The current work theoretically predicts and experimentally demonstrates a nonreciprocal spontaneous parametric process for realizing broadband optical isolation.

Conventionally, NORDs are realized with the magneto-optical effect^17,18^. Such magneto-optical devices have the unique merit of broad bandwidth, yet their compact and integrated implementation as a whole component remains an open challenge. Magnetic-free NRODs are therefore developed to tackle this challenging problem in footprint. Beside the aforementioned *unidirectionally* pumped wave mixing, various mechanisms in the classical regime have been exploited, including optical nonlinearity^19–28^, space-time phase modulation^29^, phonon-photon directional coupling^30–35^, the Sagnac effect in spinning resonators^36,37^, the macroscopic Doppler effect of unidirectionally moving Bragg optical lattice^38–40^, energy loss^41^ and directional phase matching in parametric process^42,43^. Moreover, quantum systems have also demonstrated a great success in designing novel non-magnetic NRODs by exploiting reservoir of resonators^44^, quantum nonlinearity^45^, chiral interaction of atoms and photons^46–52^, susceptibility-momentum locking^53–59^ and unidirectional quantum squeezing^60^.

In comparison with the magneto-optical counterparts, the practical applications of these magnetic-free approaches for optical isolation are often limited by their narrow bandwidth due to the requirement of high-quality resonators, or long lifetime of adopted systems. For example, the precise PMC and energy conservation in pumped wave conversion such as FWM usually allows a narrow bandwidth of tens of megahertz. However, broadband optical isolation is widely required in protecting tunable coherent laser sources, and in suppressing the backscattering noises from signals for broadband optical information processing and optical communications that support the growing data capacity, among others. Benefiting from the *broadband PMC*, a nonreciprocal spontaneous parametric process without pumping exhibits an essential advantage in potentially overcoming the limitation of bandwidth in optical nonreciprocity.

In this paper, a proof-of-concept experiment is performed in atomic vapors to show that a spontaneous parametric process driven by a weak probe laser beam can be nonreciprocal *by unidirectionally coupling it to a pumped FWM process*. Actually, coherent atomic system has served as a fertile ground in demonstrating various regimes of magnetic-free optical nonreciprocity^10,47,52–59,61^, and the achieved maximum isolation ratio is ∼40 dB with the insertion loss <1 dB^58^, but the bandwidth for isolation ratio >20 dB is limited in the level of gigahertz. Here, by employing such a nonreciprocal spontaneous parametric process in hot Na atoms as an example, we obtain optical isolation for the probe beam over 100 GHz bandwidth (corresponding to ~0.12 nm @ 589 nm) for isolation ratio >25 dB, and a maximum ratio >30 dB with the insertion loss being less than 0.7 dB. In thermal atomic ensembles, the inevitable Doppler and power-induced broadening on the decoherence rates of involved atomic energy levels are usually detrimental due to their suppression on desired atomic coherence effects. Counterintuitively, such broadening effects in our experiment extend the bandwidth of the nonlinear responses and further enable the broadband optical isolation. It’s also worth noting that the current work employs the interaction between two nonlinear processes, which is distinctive from the previous studies involving only one controlled nonlinear process such as the pumped FWM^10^. Moreover, the available nonreciprocal bandwidth in the current work is determined by the broadband property of the spontaneous parametric process, instead of the bandwidth of the pumped FWM. This fundamentally differentiates our work from previous nonreciprocal studies relying on pumped multi-wave mixing processes, whose narrow bandwidths limit the operating bandwidths of the resulting nonreciprocal behaviors.

## Results

**Fig. 1:**
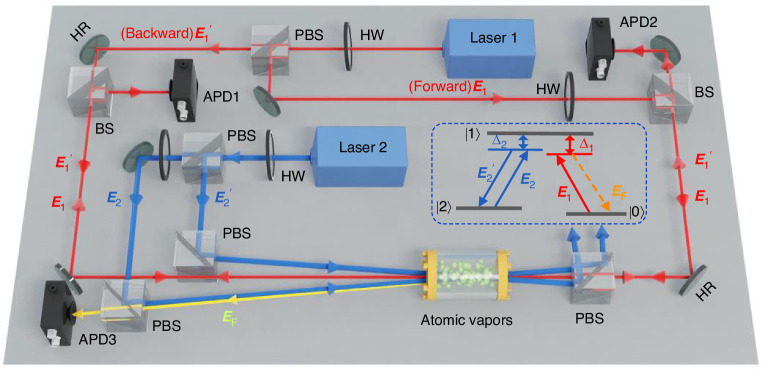
Experimental setup. The probe beams ***E***_1_ and ***E***_1_′ counter- and co-propagates with the pump beams (***E***_2_ and ***E***_2_′), respectively. The counter-propagating ***E***_1_ and two pump beams can generate the pumping four-wave mixing (pumped FWM) signal (***E***_F_), which emits along the opposite direction of ***E***_2_. The output probe and pumped FWM spectra are received by three APDs. PBS: polarization beam splitter; BS: 50/50 beam splitter; APD: avalanche photodiode detector; HW: half-wave plate; HR: high-reflectivity mirror. The inset marked by a dashed box shows the atomic configuration for the pumped FWM

Figure 1 schematically shows the experimental setup. The probe field ***E***_1_ (frequency *ω*_1_, wave vector **k**_1_, Rabi frequency *G*_1_, horizontal polarization) and two pump fields ***E***_2_ and ***E***_2_′ (*ω*_2_, **k**_2_ and **k**_2_′, *G*_2_ and *G*_2_′, vertical polarization) from two dye lasers are injected into Na atomic vapors confined in a heating pipe oven. The frequencies of the involved lasers are controlled independently by their own drivers. The energy-level configuration of atoms is depicted in the inset, involving two ground states |3S_1/2_, F = 1〉 ( | 0〉) and |3S_1/2_, F = 2〉 ( | 2〉), and an excited state |3P_3/2_〉 ( | 1〉). Two nonlinear processes happen within this coherently-prepared atomic ensemble. The probe field ***E***_1_ drives the transition |0〉→ | 1〉 and forms the four-field degenerate spontaneous parametric process, coupling to QVFs via virtual-photon process. Simultaneously, the pump fields, ***E***_2_ and ***E***_2_′, drive the transition |2〉→ | 1〉 and can excite a pumped FWM process together with the probe field.

In our experiment, the condition **k**_F_ = **k**_1_+**k**_2_−**k**_2_′ (namely PMC1) and the energy conservation (*ω*_F_ = *ω*_1_+*ω*_2_−*ω*_2_′) are simultaneously met when the probe field propagates in the opposite direction of the pump fields. In this case, the pumped FWM occurs by mediated of the probe field and a new mode *E*_F_ is generated. However, when the probe beam co-propagates with the pump beams, the pumped FWM is inefficient because the required PMC1 breaks. As a result, the probe field is decoupled from the pumped FWM process.

**Fig. 2:**
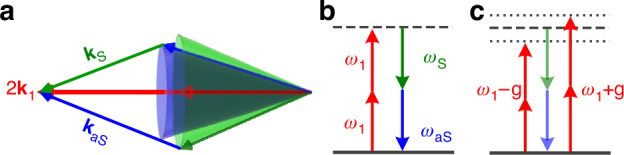
Descriptions of virtual photon process. **a** Phase-matching condition for the spontaneous parametric process under the interaction of the probe beam and sodium vapors. **b** Virtual photon process for the spontaneous parametric process when the energy conservation is met in both single probe and co-propagating cases due to the absence of pumped FWM. **c** In the counter-propagating case, ***E***_1_ is dressed by the strong coupling (under a strength of g) with ***E***_F_, and the spontaneous parametric process is suppressed due to the breaking of energy conservation

In the meanwhile, the probe field (mode *E*_1_) inside the atomic vapors can spontaneously convert to a pair of Stokes mode *E*_S_ and anti-Stokes mode *E*_aS_ (with frequencies *ω*_S_ and *ω*_aS_). Because *E*_S_ and *E*_aS_ are initially QVFs, the condition 2**k**_1_ = **k**_S_+**k**_aS_ (namely PMC2) and the energy conservation, 2*ω*_1_ = *ω*_S_+*ω*_aS_, are naturally hold over a broad bandwidth, as depicted in Fig. 2a. This is a typical spontaneous parametric process employing QVFs in a “double-Λ” atomic configuration^62^ under the dressing-state framework^63–65^, see more details in Fig. S1 in Supplementary Materials. The virtual-photon process depicting the energy conservation condition for the spontaneous parametric process is given in Fig. 2b. Conventionally, this spontaneous parametric process is independent of the propagating direction of the probe field, thus reciprocal.

However, *the inherent reciprocity can break when the probe mode directionally interacts with an auxiliary mode E*_*F*_. In the counter-propagating case, this auxiliary mode *E*_F_ enhanced by the pump fields couples to the probe mode, and the spontaneous parametric process is strongly modified by the pumped FWM. This coupling dresses *E*_1_ into energies *ω*_1_±g (see Fig. 2c) and breaks the energy conservation condition under PMC2. As the result, the spontaneous parametric process is dramatically suppressed and the probe transmission is strong. While in the co-propagating case, the pumped FWM is off so that the probe mode decouples from the *E*_F_ mode. Consequently, PMC2 retains and the spontaneous parametric process recovers efficiently. The probe field is absorbed strongly by converting to the Stokes and anti-Stokes pairs. In this sense, we create a nonreciprocal spontaneous parametric process, which can lead to different transmission characteristics for the probe beams incident from opposite directions. It’s worth mentioning that our scheme is fundamentally different from those based on parametric amplification^42^ and the unidirectionally pumped FWM^10^. In both works, the weak probe mode forms a pumped FWM with the strong pump fields under corresponding PMC and energy conservation. This pumped FWM involves a QVF with fixed momentum and energy, thus gives rise to nonreciprocity within narrow bandwidth.

The evolutions of the probe field, the paired Stokes and anti-Stokes fields during spontaneous parametric process, and the pumped FWM signal are critically related to the atomic linear and third-order nonlinear susceptibilities. The linear susceptibility is given by *χ*_*i*_^(1)^=*Nμ*_*mn*_^*2*^*ρ*_*i*_(*ω*_*i*_)/(*ε*_0_*ħG*_*i*_), where *ρ*_*i*_(*ω*_*i*_)= *iG*_*i*_/(Γ_*mn*_+*i*Δ_*i*_) is the first-order density matrix element according to the density matrix method^9^. Here we assume the atomic density *N* and consider the transition |*m*〉↔|*n*〉 (*m*, *n* = 0, 1, 2), with Γ_*mn*_ being the decoherence rate, and *μ*_*mn*_ being the electric dipole moment. The Rabi frequency of field ***E***_i_ (*i* = *aS*, *S*, 1, 2, *F*) with a complex amplitude *E*_*i*_ is described as *G*_*i*_ = *μ*_*mn*_*E*_*i*_/*ħ*. The linear absorption coefficient *γ*_*i*_ = (*ω*_*i*_/*c*)Im*χ*_*i*_^(1)^ determines the decay of the corresponding field. The nonlinear susceptibilities *χ*_*i*_^(3)^ responsible for spontaneous parametric process and pumped FWM are positively dependent on the corresponding third-order density matrix elements *ρ*_*i*_^(3)^ (see Supplementary Materials), which are described as:

1.1 $${\rho }_{s}^{(3)}=-i{G}_{1}^{2}{G}_{aS}^{\ast }/[({d}_{1}+{G}_{{{F}}}^{2}/{\varGamma }_{00E}){d}_{0}({d}_{1}+i{\delta }_{1}+{G}_{{{F}}}^{2}/{d}_{0})]$$

1.2 $${\rho }_{as}^{(3)}=-i{G}_{1}^{2}{G}_{S}^{\ast }/[({d}_{1}+{G}_{{{F}}}^{2}/{\varGamma }_{00E}){d}_{2}({d}_{1}-i{\delta }_{1}+{G}_{{{F}}}^{2}/{d}_{2})]$$

1.3 $${\rho}_{F}^{(3)}=-i{G}_{1}{|{G}_{2}|}^{2}/\{{d}_{1}({\varGamma}_{21E}+i{\Delta} ^{\prime}_{2})[{\varGamma }_{20E}+i({\Delta}_{1}-{\Delta}^{\prime}_{2})]\}$$

where *d*_0_ = *Γ*_00*E*_ + *iδ*_1_, *d*_1_ = *Γ*_10*E*_ + *i*Δ_1_, and *d*_2_ = *Γ*_00*E*_−*iδ*_1_, with *δ*_1_ being the frequency gap of two-dressing states induced by ***E***_1_. The probe detuning Δ_1_ = *ω*_10_−*ω*_1_ is defined as the difference between the frequency interval of the levels connected by ***E***_1_ and its frequency. The pump detuning Δ_2_′ satisfies Δ_1_−Δ_2_′ = 0, describing the two-photon resonant condition for the pumped FWM. See more details of dressing states in Fig. S1 in Supplementary Materials. The natural decoherence rate Γ_mn_ for single Na atom without interacting with light is about tens of megahertz. For laser-excited thermal atomic ensembles, the Doppler and power-induced broadening of the decoherence rate need to be considered^64^. In light-induced atomic coherent processes, the driving laser can broaden the distribution of the energy levels it connects, and its power determines the degree of such broadening. For example, the linewidth of the well-known electromagnetically induced transparency (EIT) can increase with the intensity of the pump field^66^. As a consequence, the effective decoherence rate Γ_mn*E*_ becomes tens of gigahertz (see Supplementary Materials). The power and Doppler broadening effects together make great contributions to expanding the valid range of bandwidth for the nonreciprocal spontaneous parametric process.

The spontaneous parametric process and pumped FWM process are governed by the following effective interaction Hamiltonian between field modes^9^:

2 $${H}_{I}=i\hslash [({\kappa }_{S}+{\kappa }_{aS})/2]{\hat{a}}_{1}^{2}{\hat{a}}_{S}^{\dagger }{\hat{a}}_{aS}^{\dagger }+i\hslash {\kappa }_{F}{\hat{a}}_{2}{\hat{a}{^{\prime}}}_{2}^{\dagger }{\hat{a}}_{F}^{\dagger }{\hat{a}}_{1}+H.c.{\,}$$

where *â*_*i*_ (*i* = *aS*, *S*, 1, 2, *F*) is the bosonic annihilation operator for corresponding field, the operator *â*_2_′ is for ***E***_2_′, and the coefficient *κ*_*i*_ = |−*ik*_*i*_*χ*_*i*_^(3)^| represents the coupling strength depending on *χ*_*i*_^(3)^. In the limitation of the strong classical fields, we can replace the annihilation operator *â*_*i*_ with the complex amplitude *E*_*i*_ of the positive-frequency component of the corresponding field.

From the nonlinear Helmholtz equation under the slowly-varying amplitude approximation^9,53^, we obtain the coupled equations describing the propagation of spontaneous parametric process and pumped FWM as:

3.1 $$\partial {E}_{1}/\partial z=-{\gamma }_{1}{E}_{1}-({\kappa }_{S}+{\kappa }_{aS}){E}_{1}^{\ast }{E}_{S}{E}_{aS}\times M-{\kappa }_{F}{|{E}_{2}|}^{2}{E}_{F}$$

3.2 $$\partial {E}_{s}/\partial z=-{\gamma }_{S}{E}_{S}+{\kappa }_{aS}{|{E}_{1}|}^{2}{E}_{aS}^{\ast }\times M+{\beta }_{S}$$

3.3 $$\partial {E}_{aS}/\partial z=-{\gamma }_{aS}{E}_{aS}+{\kappa }_{S}{|{E}_{1}|}^{2}{E}_{S}^{\ast }\times M+{\beta }_{aS}$$

3.4 $$\partial {E}_{F}/\partial z=-{\gamma }_{F}{E}_{F}+{\kappa }_{F}{|{E}_{2}|}^{2}{E}_{1}$$

with $${\beta }_{i}=\sqrt{2{\gamma }_{i}}{\hat{\xi }}_{i}(z)$$ being the Langevin noise modeling the quantum fluctuation^67–69^, which is important for generating spontaneous parametric process. The noise operator satisfies the correlation functions $$\langle {\hat{\xi }}_{i}^{\dagger }(z){\hat{\xi }}_{i}(z^{\prime} )\rangle =0$$ and $$\langle {\hat{\xi }}_{i}(z){\hat{\xi }}_{i}^{\dagger }(z^{\prime} )\rangle$$$$=\delta (z-z^{\prime} )$$. Here, *κ*_F_ = 0 is for the co-propagating case while the nonzero *κ*_F_ for the counter-propagating case. Because the pump fields ***E***_2_ and ***E***_2_′ are strong and propagate in almost the same direction, we consider them constant during propagation. For simplicity, we replace *E*_2_′ with *E*_2_ due to their identical intensity. In experiment, the Stokes and anti-Stokes fields can be generated in pair in the spontaneous parametric process over a wide range of the frequency and wave vectors, exhibiting a cone (containing hundreds of paired spatial modes) around the probe beam^70–72^. To consider this effect, we multiple the spontaneous parametric signals by a factor M. *E*_S_ and *E*_*a*S_ in Eq. (3) define each pair of Stokes and anti-Stokes modes.

**Fig. 3:**
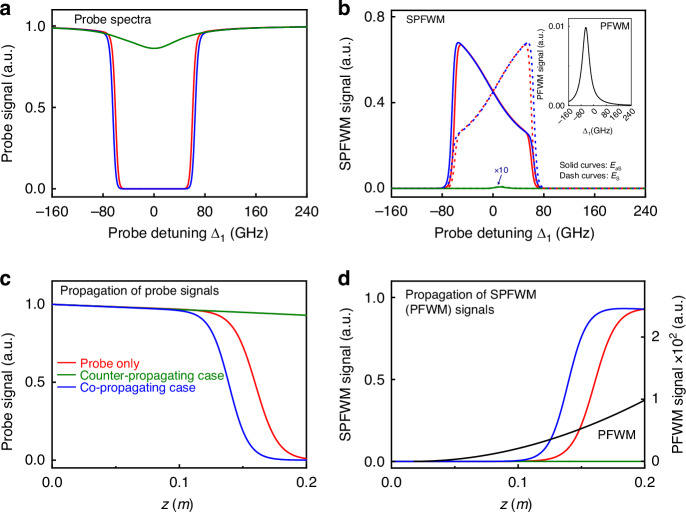
Simulated output spectra and evolution of involved fields in atomic vapors in probe only (red), co-propagating (backward, blue), and counter-propagating (forward, green) cases. All intensities are normalized by the input intensity of the probe beam. **a** Spectra of the probe field. **b** Spectra of the spontaneous parametric and pumped FWM (inset) signals at Δ_2_′=−2π × 8.2 GHz. **c** Evolutions of the probe field. **d** Evolutions of the spontaneous parametric signal at Δ_1_ = Δ_2_′ = −2π × 8.2 GHz. The propagation of pumped FWM signal (black curve) is also provided for the forward case. Other parameters: *N* = 1.24 × 10^14^ cm^−3^ (the forward case) and 1.43 × 10^14^ cm^−3^ (the backward case), *G*_1_=2π × 6 GHz, *G*_2_=*G*_2_′= 2π × 30 GHz, and M = 600

Figure 3a and b show the simulated spectra of the transmitted probe field, the Stokes and anti-Stokes fields, and pumped FWM signals after passing the atomic vapors for three cases: (i) in the absence of the pump beams (probe only, red curves); (ii) the co-propagating case (backward, blue curves); (iii) the counter-propagating case (forward, green curves). The spontaneous parametric process is efficient in cases (i) and (ii), and the probe field is completely expended over a broad bandwidth [see red and blue curves in Fig. 3a] due to the resonant absorption and the considerable conversion to spontaneous parametric signals. In the backward case (ii), the pumped FWM process annihilates because PMC1 breaks, and its interaction with the probe beam is negligible. As a result, the generations of paired spontaneous parametric signals [red and blue curves in Fig. 3b] are similar in the two cases, so are the transmissions of the probe field. Nevertheless, the spontaneous parametric signals are slightly stronger and the probe transmission is accordingly lower in case (ii) than that in case (i). This is because the optical pumping effect of the introduced pump fields increases the effective atomic density^56,73^, leading to a more efficient spontaneous parametric process. Here the anti-Stokes and Stokes modes occur symmetrically with respect to Δ_1_=0, since they are different in frequency. Figure 3c theoretically shows the propagation of the probe beam inside the medium. In both cases, the probe first declines slowly when the Stokes and anti-Stokes signals are weak. Then, it decays from *z*=0.13 m (*z*=0.12 m) rapidly to vanishing small at *z*=0.2 m (*z*=0.16 m) in the probe only (backward) case. Correspondingly, the generated spontaneous parametric signals increase fast to a saturated intensity during this propagation distance, see Fig. 3d.

In obvious contrast with the backward case (ii), the probe in the forward case (iii) shows a strong nonreciprocal transmission. The pumped FWM process occurs because of the satisfaction of PMC1 and energy conservation. This is reflected by the pumped FWM signals (black curves) shown in Fig. 3b, d. The significance of the occurred pumped FWM lies in modulating the spontaneous parametric process by introducing a strong coupling between the probe mode and the *E*_F_ mode. This chiral coupling interaction dresses the probe energy and unidirectionally breaks the energy conservation condition for spontaneous parametric process. Therefore, the spontaneous parametric process is greatly suppressed and the probe field can pass through atomic vapors with small attenuation, see Fig. 3a, c, indicated by the very weak spontaneous parametric signal (green curve) in Fig. 3d. The nonreciprocal spontaneous parametric process is in coincidence with the calculated third-order susceptibilities for three cases (Fig. S2 in Supplementary Materials).

**Fig. 4:**
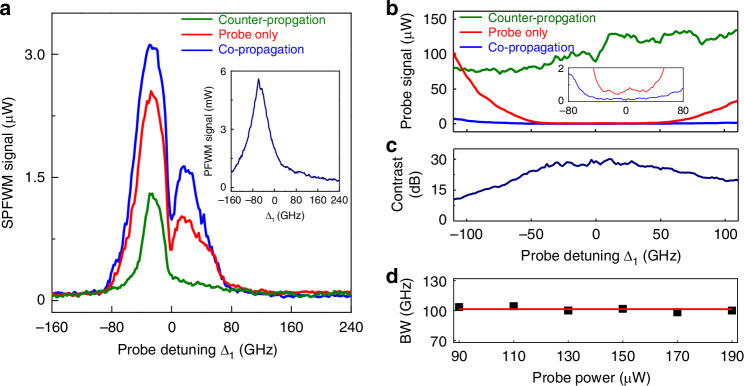
Experimental observations. **a** Measured spectra of spontaneous parametric signals and **b** transmitted probe as a function of the probe detuning for only a unidirectional probe beam applied (red), the co-propagation case (blue) and counter-propagation case (green). The inset in **a** shows the observed pumped FWM spectrum. **c** Nonreciprocal transmission contrast calculated from **b**. **d** Bandwidth (BW) for isolation ratio >25 dB versus the probe power when two probe beams (***E***_1_ and ***E***_1_′ in Fig. 1) propagate simultaneously in opposite directions. The square are experimental data, while the solid line represents a fitting and provides a guide to the eye. The probe power is 150 μW in (**a**–**c**). The power of both pump beams are 3.75 mW

The nonreciprocity of the spontaneous parametric process is experimentally displayed in Fig. 4a. Considering the spatial ring structure of the spontaneous parametric process, the anti-Stokes and Stokes modes are undistinguishable in space^62^. The captured spectrum of spontaneous parametric process contains both modes, and exhibits a double-peak profile, corresponding to the sum of anti-Stokes and Stokes components (see theoretical Fig. S3 in Supplementary Materials). Here we measure the power spectra of the Stokes and anti-Stokes modes generated in the spontaneous parametric process in aforementioned three corresponding cases. The spontaneous parametric process in the cases (i) and (ii) are strong because the pumped FWM process is prevented and the probe mode decouples from the *E*_F_ mode. Consequently, the probe field converts to the Stokes and anti-Stokes modes efficiently, see red and blue curves in Fig. 4a, and the corresponding probe transmissions are weak (see Fig. 4b). Since the strong pump fields repump the atoms, the conversion is enhanced and the transmission is lower in the backward case (ii). In distinct contrast, the pumped FWM happens in the forward case (iii) and heavily interacts with the probe mode. This interaction modifies the probe mode to break the PMC2 for spontaneous parametric process over a wide range of frequency. Therefore, the spontaneous parametric process is substantially suppressed (green curve in Fig. 4a) and the corresponding probe transmission is high (green curve in Fig. 4b).

The intensity of the observed anti-Stokes and Stokes spectra exhibits an asymmetric structure with respect to Δ_1_ = 0, due to the other transition |3S_1/2_〉→ |3P_1/2_〉 of Na atoms. Considering the Doppler and power-induced broadening effect on the state |3P_1/2_〉, the incident ***E***_1_ (resonant with |3S_1/2_〉→ | 3P_3/2_〉) can also excite the transition |3S_1/2_〉→ |3P_1/2_〉, but under the far-detuning condition. Namely, both transitions can produce absorption for ***E***_1_ and spontaneous parametric process. A smaller value of Δ_1_ means that frequency of probe field is set further from the resonance of state |3P_1/2_〉, which hence gives rise to a weaker absorption. The observed asymmetric intensity profile of the probe transmission (probe only case) advocates this effect (Fig. S4 in Supplementary Materials). When the absorption from the transition 3S_1/2_→3P_1/2_ is theoretically considered, the simulated spontaneous parametric spectrum exhibits an asymmetric double-peak profile (Fig. S3 in Supplementary Materials). In the meanwhile, the self-Kerr nonlinearity also contributes to the asymmetric profile. For Δ_1_ < 0 and Δ_1_ > 0 regions, the nonlinear atomic system can behave focusing and defocusing, respectively, due to the opposite signs of self-Kerr nonlinearity^74,75^. The spot size of the probe in the defocusing case is broader than that in the focusing case. The beam with a larger size covers and interacts with more atoms, thus enabling stronger resonant absorption, which indicates that the probe can provide less energy and produce weaker spontaneous parametric signals, resulting in more asymmetric spectra in experiment.

The transmitted probe intensities of the forward and backward cases are denoted as *T*_*f*_ and *T*_*b*_, and we calculate the isolation ratio as *η*=10log_10_(*T*_*f*_/*T*_*b*_). The contrast dependence on the probe detuning is presented in Fig. 4c with either probe laser beam (***E***_1_ or ***E***_1_′ in Fig. 1) selectively on. The contrast is larger than 25 dB over 100 GHz band (approximately −45 GHz to 55 GHz) and reaches the maximum value 30 dB (corresponding an insertion loss of <0.7 dB) at Δ_1_≈10 GHz. According to Eq. (1), the further enhancement of the pump-field intensity will produce a stronger pumped FWM, which can provide more suppression on the Stokes and anti-Stokes signals to increase the forward probe transmission. In addition, a stronger pump field can lead to more efficient optical pumping effect^76^ to reduce the backward probe transmission. Such a combined effect in both backward and forward cases will undoubtedly improve the isolation ratio and the insertion loss. However, limited by our experimental condition, the accessible maximum output power of both pump beams is 3.75 mW.

Our nonreciprocal regime can bypass dynamic reciprocity^77^, which impose severe constraint on most nonlinear isolators, when two probe beams are oppositely (collinear) incident into the medium together. The transmission contrast versus the probe detuning is close to the single-probe contrast shown in Fig. 4c, according to Fig. S5 in Supplementary Materials. The nonreciprocal bandwidth for contrast >25 dB exceeds 100 GHz and remains stable as the input power increases from 90 μW to 190 μW (see Fig. 4d). This result indicates that the demonstrated optical isolation exhibits robustness against the input probe power over a broad bandwidth. The nonreciprocal behavior with both probe fields present also advocates the interaction between ***E***_F_ and the spontaneous parametric process is nonreciprocal. This is due to the Doppler frequency shifts caused by the unavoidable random thermal motion of atoms^53,56^.

## Discussion

In summary, we experimentally demonstrated a non-reciprocal spontaneous parametric process by breaking its spatial inversion symmetry and time reversal symmetry after introducing a pumped FWM. Based on this nonreciprocal spontaneous parametric process, we achieved optical isolation for the injected probe field with large isolation ratio and broad bandwidth. The development of chip-scale vapor cells^78,79^ that are compatible with wafer-scale fabrication will be promisingly expected to promote the miniaturization and integration of the designed nonreciprocal spontaneous parametric configuration. Further, considering that both spontaneous parametric process and pumped FWM processes have been realized in not only gaseous but also diverse solid optical platforms. The concept of this work can promising motivate the explorations of integrated broadband optical nonreciprocity by employing the interactions between commonly seen nonlinear optical processes in solid nonlinear materials. Also, the demonstrated nonreciprocal spontaneous parametric process with quantum nature may also stir up the studies in designing broadband nonreciprocal quantum devices.

## Materials and methods

Both dye lasers are pumped by the same injection-locking single-mode Nd: YAG pulse laser (Continuum Powerlite DLS 9010, 10 Hz repetition rate, 5 ns pulse width). The wavelength of the probe laser is set as ~589.0 nm, while the coupling laser works around ~589.6 nm. The angle between the two pump beams are about ~0.3°. The output of three APDs enter a multi-channel SR200 series Boxcar Averager system (followed by a data acquisition setup) to plot the spectra signals. The temperature of the sodium atomic vapors inside the heating pipe oven (with a length of 20 cm) is about 280 °C, corresponding to an atomic density of ~1.24 × 10^14 ^cm^−3^.

## Supplementary information

Supplemental Materials for Nonreciprocal Spontaneous Parametric Process

## Data Availability

The experimental data generated in this study are openly available in the Open Science Framework (OSF) at 10.17605/OSF.IO/9RKQU, under a CC-BY 4.0. license.
