# Supplementary material for: Nonreciprocal spontaneous parametric process
Source: Light Sci Appl. 2025 May 19;14:200. doi: 10.1038/s41377-025-01844-8 (PMC12086202; doi:10.1038/s41377-025-01844-8)
Supplement: Supplementary file 1 — Supplemental Materials for Nonreciprocal Spontaneous Parametric Process [file 41377_2025_1844_MOESM1_ESM.pdf]

Supplemental Materials for

Nonreciprocal spontaneous parametric process

Changbiao Li[1,*], Jiaqi Yuan[1,*], Ruidong He[1], Jiawei Yu[1], Yanpeng Zhang[1], Min Xiao[2,3], Keyu Xia[3†] and Zhaoyang Zhang[1,‡]

[1]*Key Laboratory for Physical Electronics and Devices of the Ministry of Education & Shaanxi Key Lab of Information Photonic Technique, School of Electronic Science and Engineering, Faculty of Electronic and Information Engineering, Xi'an Jiaotong University, Xi'an, 710049, China*

[2]*Department of Physics, University of Arkansas, Fayetteville, Arkansas, 72701, USA*

[3]*College of Engineering and Applied Sciences, National Laboratory of Solid State Microstructures, and School of Physics, Nanjing University, Nanjing 210093, China*

[*]These authors contributed equally to this work.

Corresponding authors: [†]keyu.xia@nju.edu.cn, [‡]zhyzhang@xjtu.edu.cn

I. Dressing-state pictures in a three-level Na atoms

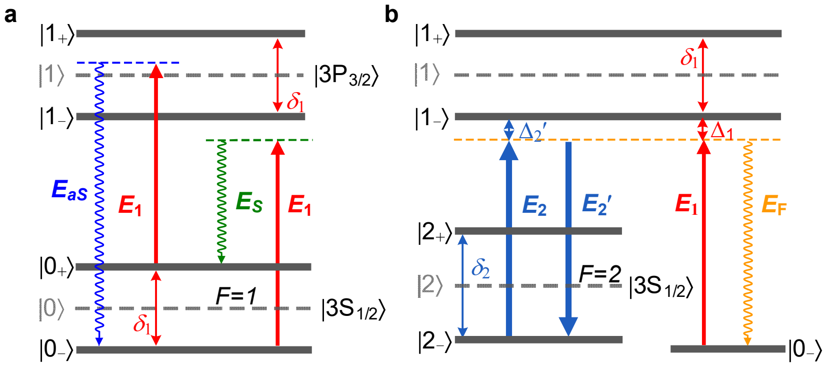

Fig. S1 a Double-Λ atomic energy-level configuration for the spontaneous parametric process. The probe detuning $\Delta_1=\omega_{10}-\omega_1$ is defined as the difference between the transition frequency of the two original energy levels [the ground state $|3S_{1/2}, F=1\rangle$ ($|0\rangle$) and the excited state $|3P_{3/2}\rangle$ ($|1\rangle$)] and the frequency of $\boldsymbol{E}_1$. The states $|0_+\rangle$ and $|0_-\rangle$ ($|1_+\rangle$ and $|1_-\rangle$) with a frequency difference of δ_1 represent two dressed states of the ground level $|0\rangle$ (the excited level $|1\rangle$) induced by $\boldsymbol{E}_1$. **b** Three-level atomic energy-level configuration for the pumped FWM. The states $|2_+\rangle$ and $|2_-\rangle$ with frequency difference of δ_2 are two dressed states of another ground level $|2\rangle$ ($|3S_{1/2}, F=2\rangle$) induced by pump beams. The pump detuning is defined as $\Delta_2=\omega_{12}-\omega_2$.

Figure S1**a** depicts the energy-level configuration for the spontaneous parametric process and pumped four-wave mixing (FWM) under the dressing-state framework. The probe field $\boldsymbol{E}_1$ (frequency ω_1, wave vector $\mathbf{k}_1$, Rabi frequency G_1) drives transition $|3S_{1/2}\rangle\rightarrow|3P_{3/2}\rangle$ and enables the spontaneous parametric process to generate the Stokes and anti-Stokes modes ($\boldsymbol{E}_S$ and $\boldsymbol{E}_{aS}$) via a virtual photon process. The "double-Λ" atomic configuration involves the dressing states $|0_\pm\rangle$ of the ground state $|0\rangle$ and $|1_\pm\rangle$ of the excited state $|1\rangle$. According to the dressing-state picture[1], the probe beam can simultaneously split states $|0\rangle$ and $|1\rangle$ into new virtual states $|0_\pm\rangle$ and $|1_\pm\rangle$, respectively,

with the same frequency gap of $\delta_1=\lambda_{0+}-\lambda_{0-}=(\Delta_1^2+G_1^2)^{1/2}$, where $\lambda_{0\pm}$ $(=\lambda_{1\mp})$ $=\Delta_1/2\pm(\Delta_1^2+G_1^2)^{1/2}/2$ are the eigenvalues of states $|0_\pm\rangle$ $(|1_\mp\rangle)$[2,3]. The frequencies of the conjugated modes $\boldsymbol{E}_{aS}$ and $\boldsymbol{E}_S$ are $\omega_1\pm\delta_1$, respectively, indicating energy conservation. We define the detuning as $\Delta_{aS}=\omega_{10}-\omega_{aS}=\Delta_1-\delta_1$ and $\Delta_S=\omega_{10}-\omega_S=\Delta_1+\delta_1$. When only the probe field propagates in the atomic vapor (the probe-only case), the spontaneous parametric process occurs with the same efficiency for the opposite-input probe fields, thus is reciprocal.

In the configuration shown in Fig. S1**b**, the probe and pump fields $\boldsymbol{E}_2$ and $\boldsymbol{E}_2'$ (ω_2, $\mathbf{k}_2$ and $\mathbf{k}_2'$, G_2 and G_2') excite a pumped FWM process with wave vector $\mathbf{k}_F$ and frequency ω_F. Here the pump beams also create two dressing states $|2_\pm\rangle$ (separating by $\delta_2=\lambda_{2+}-\lambda_{2-}$) with eigenvalues being $\lambda_{2\pm}=\Delta_2/2\pm(\Delta_2^2+G_2^2+G_2'^2)^{1/2}/2$. The two-photon resonant condition for the pumped FWM is $\Delta_1-\Delta_2'=0$ with $\Delta_2'=\Delta_2-\lambda_{1+}+\lambda_{2-}$, considering different eigenvalues for dressing states caused by the probe and pump beams.

II. Doppler- and power-induced broadening in the thermal atomic ensemble

The natural decoherence rate Γ_{mn} for single Na atom without experiencing the interaction with light is about tens of megahertz. For thermal atomic ensembles, the Doppler- and power-induced broadening of the decoherence rate need to be considered. The effective decoherence rate Γ_{mnE} in the presence of $\boldsymbol{E}_1$ becomes $\Gamma_{mnE}=\Gamma_D+\Gamma_P$. For a given Rabi frequency G_i, the power broadening can be estimated as $\Gamma_P=\Gamma_{mn}(1+G_i^2/\Gamma_{mn}^2)^{1/2}$ [3]. The Doppler broadening is ~11 GHz according to $\Gamma_D=2(\ln 2)^{1/2}u\omega_i/c$ with $u=(2K_BT/w)^{1/2}$, where w is the mass of a Na atom, K_B is the Boltzmann's constant, and T is the absolute temperature. When only $\boldsymbol{E}_1$ is on with a Rabi frequency of 2π×6 GHz, the effective Γ_{10E} (for simulating spontaneous parametric process) is about 48.7 GHz (Γ_P=37.7 GHz with Γ_{10}=61.5 MHz).

III. Calculated third-order susceptibility for Stokes and anti-Stokes signals

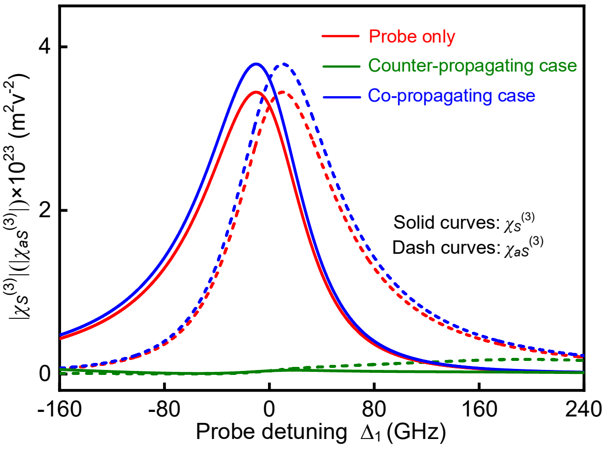

Fig. S2 The simulated susceptibilities of Stokes (solid curves) and anti-Stokes (dash curves) signals versus the probe detuning (with Δ_2'=−2π×8.2 GHz) under the conditions of single probe beam (red),

counter-propagating case (green), and co-propagating case (blue). The adopted parameters are: N=1.24×10^{14} cm^{-3} (counter-propagating case) and 1.43×10^{14} cm^{-3} (co-propagating case), G_1=2π×6 GHz, G_2=2π×20 GHz.

The third-order susceptibilities related to spontaneous parametric process and pumped FWM are given by $\chi_{aS/S}^{(3)}=|N\mu_{10}\rho_{aS/S}^{(3)}/(\varepsilon_0 E_1^2 E_{s/aS})|$ and $\chi_F^{(3)}=|N\mu_{10}\rho_F^{(3)}/(\varepsilon_0 E_1 E_2^2)|$, respectively. According to the definitions of the third-order density matrix elements $\rho_i^{(3)}$ in Eqs. (1.1) and (1.2) in the main text, we simulate the susceptibilities of the generated Stokes and anti-Stokes signals in Fig. S2. One can see that for probe only, co- and counter-propagating cases, the profiles of susceptibility for the Stokes and anti-Stokes modes distribute symmetrically along Δ_1=0. The peak values correspond to the frequencies of Stokes and anti-Stokes signals.

IV. Theoretical intensity spectra of spontaneous parametric process

Considering the undistinguishable property of the anti-Stokes and Stokes modes in space, we simulate the supposition of the Stokes and anti-Stokes modes given in Fig. 3 (in the manuscript), and the resulted spectral profile shows a double-peak profile (gray solid curve in Fig. S3) as expected, and the intensity is symmetric with respect to Δ_1=0.

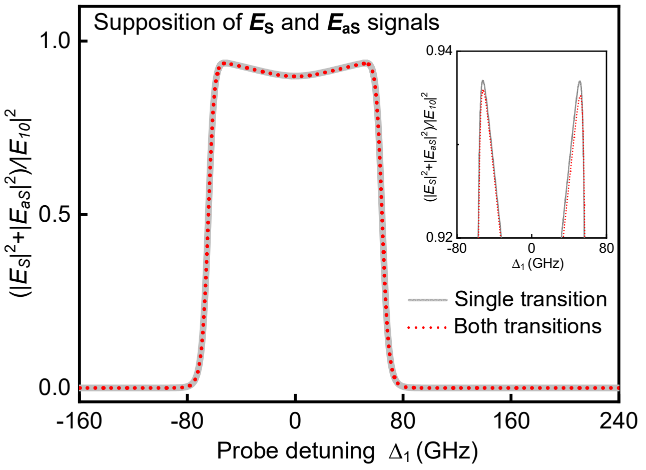

Fig. S3 The simulated supposition of Stokes and anti-Stokes signals versus the probe detuning when the probe and the pump beams are in the co-propagating situation. The gray solid (red dotted) curve represents the sum of Stokes and anti-Stokes modes without (with) considering the absorption from the transition $|3S_{1/2}\rangle\rightarrow|3P_{1/2}\rangle$. We zoom in the double-peak profile in the inset. The parameters are the same as that of co-propagating case in Fig. S2.

With the absorption from the transition $|3S_{1/2}\rangle\rightarrow|3P_{1/2}\rangle$ taking into consideration, the peak at Δ_1>0 shows weaker intensity than that at Δ_1<0 (as given by red dotted curve and inset in Fig. S3), agreeing with the observed spontaneous parametric process spectrum exhibiting an asymmetric intensity profile. Because both transitions $|3S_{1/2}\rangle\rightarrow|3P_{1/2}\rangle$ and $|3S_{1/2}\rangle\rightarrow|3P_{3/2}\rangle$ are excited, here we should modify the linear

susceptibility as $\chi'^{(1)}_i = -N\mu_{mn}^2 \rho_i'(\omega_i)/(\varepsilon_0 \hbar G_i)$ with $\rho_i'(\omega_i) = iG_i/(\Gamma_{mnE}+i\Delta_i)+iG_i/(\Gamma+i\Delta_i')$, where $\Gamma \approx \Gamma_{mnE}$ is the decoherence rate and $\Delta_i' = \omega - \omega_i$, with ω being resonant frequency for $|3S_{1/2}\rangle \to |3P_{1/2}\rangle$ and ω_i ($i=aS$, S, 1) being the frequency of field $\boldsymbol{E}_i$.

V. Observed probe transmission and spontaneous parametric signals

Figure S4 experimentally shows the transmitted probe spectrum with strong loss around the single-photon resonance $\Delta_1=0$ when only single probe beam is incident, and the corresponding spontaneous parametric process occurs in the approximate range of -100 GHz$\le\Delta_1\le$100 GHz. The intensity attenuation of the probe beam at detuning far away from the resonance (beyond the range of spontaneous parametric process) is due to the absorptive nature of alkalis atoms. Here the asymmetric profiles of the probe and spontaneous parametric process spectra are caused by the additional absorption from the transition $|3S_{1/2}\rangle \to |3P_{1/2}\rangle$. Besides, the focusing ($\Delta_1<0$) and defocusing ($\Delta_1>0$) effects due to the self-Kerr nonlinearity also contribute to the asymmetric profile. The broader (narrower) spot size of the probe in the defocusing (focusing) case makes its cover and interact with more (less) atoms, thus enabling stronger (weaker) resonant absorption.

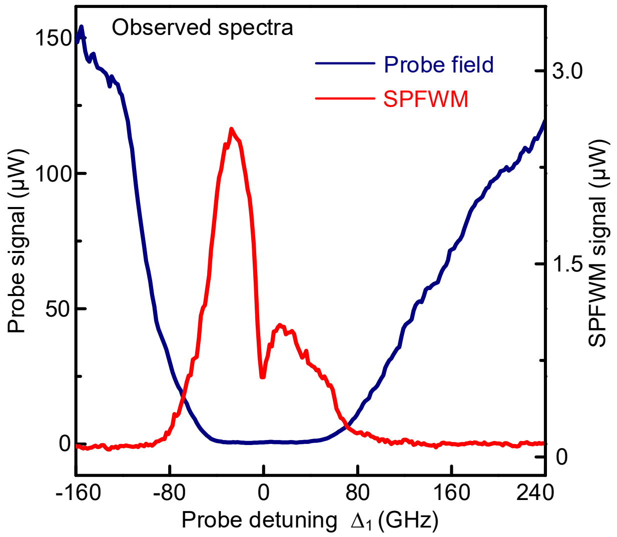

Fig. S4 Observed output spectra of probe beam and corresponding spontaneous parametric signal versus the probe detuning with only single probe beam injected.

VI. Evolutions of the isolation performance on the probe power

Figure S5 experimentally shows the evolution of the isolation performance with both the forward and backward probe beams present by simultaneously increasing their power from 90 μW to 190 μW. The observed forward and backward transmission spectra at different probe powers are given in Figs. S5**a** and S5**b**, respectively. The corresponding isolation ratio are calculated in Fig. S5**c**. One can see that the maximum

contrast is always over 30 dB and the region with contrast over 25 dB keeps almost unchanged.

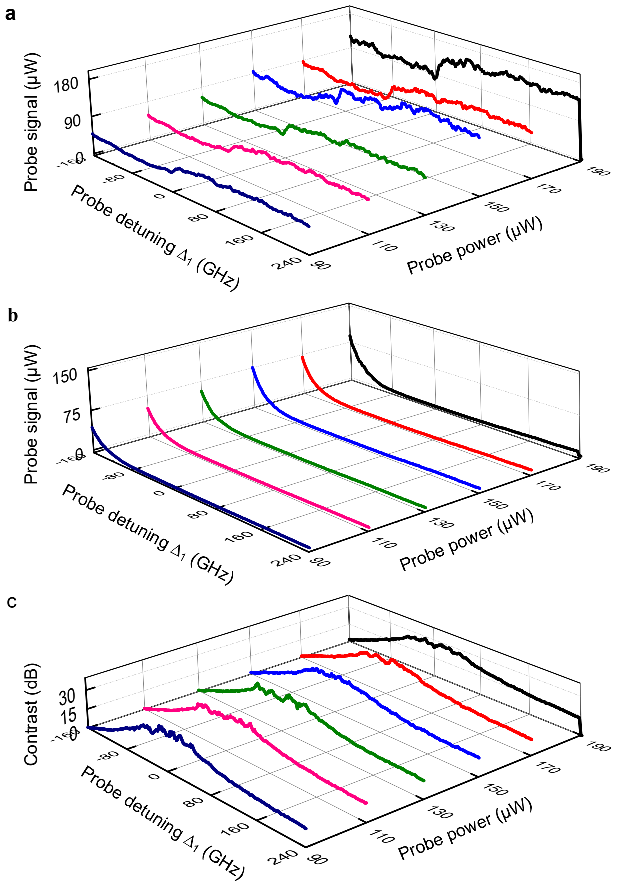

Fig. S5 a and **b** give the probe transmission spectra (with both probe beams on) in the forward and backward cases, respectively, at different probe powers. **c** Corresponding isolation ratios. The parameters for pump beams are the same as that in Fig. 4 in the manuscript.

References

1. Barnett, S. M. & Radmore, P. M. Methods in theoretical quantum optics, London: Oxford University Press (2002).
2. Krems, R. V. Molecules in electromagnetic fields, New York: John Wiley & Sons, Inc (2019).
3. Sun, J. et al. Two-photon resonant four-wave mixing in a dressed atomic system, *Phys. Rev. A* **70**, 053820 (2004).
